# Association of Periconception Paternal Body Mass Index With Persistent Changes in DNA Methylation of Offspring in Childhood

**DOI:** 10.1001/jamanetworkopen.2019.16777

**Published:** 2019-12-27

**Authors:** Nudrat Noor, Andres Cardenas, Sheryl L. Rifas-Shiman, Hui Pan, Jonathan M. Dreyfuss, Emily Oken, Marie-France Hivert, Tamarra James-Todd, Mary-Elizabeth Patti, Elvira Isganaitis

**Affiliations:** 1 Department of Environmental Health, Harvard T.H. Chan School of Public Health, Boston, Massachusetts; 2 Department of Epidemiology, Harvard T.H. Chan School of Public Health, Boston, Massachusetts; 3 Division of Chronic Disease Research Across the Lifecourse, Department of Population Medicine, Harvard Medical School and Harvard Pilgrim Health Care Institute, Boston, Massachusetts; 4 Division of Environmental Health Sciences, School of Public Health, University of California, Berkeley; 5 Research Division, Joslin Diabetes Center, Boston, Massachusetts; 6 Department of Nutrition, Harvard T.H. Chan School of Public Health, Boston, Massachusetts; 7 Department of Medicine, Harvard Medical School, Boston, Massachusetts; 8 Department of Pediatrics, Harvard Medical School, Boston, Massachusetts

## Abstract

**Importance:**

While prenatal nutrition and maternal obesity are recognized as important contributors to epigenetic changes and childhood obesity, the role of paternal obesity in the epigenome of offspring has not been well studied.

**Objectives:**

To test whether periconception paternal body mass index (BMI) is associated with DNA methylation patterns in newborns, to examine associations between maternal and paternal BMI and the epigenome of offspring, and to examine persistence of epigenetic marks at ages 3 and 7 years.

**Design, Setting, and Participants:**

Project Viva is a prebirth cohort study of mothers and children including 2128 live births that enrolled mothers from April 1999 to July 2002 and followed offspring to adolescence. This study analyzed the subset of participants with available data on paternal BMI and DNA methylation in offspring blood in the newborn period, at age 3 years, and at age 7 years. Data were analyzed from July 2017 to October 2019.

**Exposures:**

The primary exposure was paternal periconception BMI; associations were adjusted for maternal prepregnancy BMI and stratified according to maternal BMI above or below 25.

**Main Outcomes and Measures:**

The primary outcome was genome-wide DNA methylation patterns in offspring blood collected at birth, age 3 years, and age 7 years.

**Results:**

A total of 429 father-mother-infant triads were included. The mean (SD) periconception paternal BMI was 26.4 (4.0) and mean maternal prepregnancy BMI was 24.5 (5.2); 268 fathers had BMI greater than or equal to 25 (mean [SD], 28.5 [3.3]) and 161 had BMI less than 25 (mean [SD], 22.8 [1.8]). Paternal BMI greater than or equal to 25 was associated with increased offspring birth weight compared with paternal BMI less than 25 (mean [SD] *z* score, 0.38 [0.91] vs 0.11 [0.96]; *P* = .004). Cord blood DNA methylation at 9 CpG sites was associated with paternal BMI independent of maternal BMI (*q* < .05). Methylation at cg04763273, between *TFAP2C* and *BMP7*, decreased by 5% in cord blood with every 1-unit increase in paternal BMI (*P* = 3.13 × 10^−8^); hypomethylation at this site persisted at ages 3 years and 7 years. Paternal BMI was associated with methylation at cg01029450 in the promoter region of the *ARFGAP3* gene; methylation at this site was also associated with lower infant birth weight (β = −0.0003; SD = 0.0001; *P* = .03) and with higher BMI *z* score at age 3 years.

**Conclusions and Relevance:**

In this study, paternal BMI was associated with DNA methylation, birth weight, and childhood BMI *z* score in offspring.

## Key Points

**Question:**

Is paternal obesity associated with epigenetic marks and weight status in offspring?

**Findings:**

This cohort study of 429 father-mother-infant triads found that paternal body mass index at the time of conception was associated with both offspring birth weight and epigenome-wide DNA methylation patterns in offspring at birth, age 3 years, and age 7 years.

**Meaning:**

These findings suggest that paternal obesity may be an underappreciated contributor to childhood health outcomes.

## Teaser

This cohort study investigates the associations between periconception paternal body mass index (BMI) and DNA methylation patterns in offspring.

## Introduction

Nutritional and environmental exposures during early development are associated with long-term health and disease.^1,2^ Thus, optimizing maternal health and nutrition prior to conception and during pregnancy is a major focus for population health and prevention efforts.^3^ Emerging evidence now suggests that not only maternal, but also paternal, exposures may influence disease patterns and obesity risk in offspring. Our group and others^4,5,6^ have reported that paternal undernutrition in rodents is associated with increased risk of obesity, diabetes, and hepatic steatosis in offspring. Additional paternal exposures, including low-protein diet, high-fat diet, hyperglycemia, psychological stress, and endocrine-disrupting chemicals, are also associated with behavioral responses, obesity, and diabetes risk in progeny in animal models.^6,7,8,9,10,11,12,13^ While epigenetic factors in male germ cells are thought to contribute, the precise mechanisms for nongenetic, paternal inheritance remain incompletely defined.

Recent clinical studies have shown that obesity and weight loss may alter DNA methylation and expression of noncoding RNA in sperm, raising the possibility that paternally transmitted epigenetic inheritance could occur in human populations.^14^ Obesity-associated epigenetic changes in sperm have been reported at imprinted genes^15^ and at genomic loci linked to appetite and weight regulation.^14^ Whether these obesity-associated epigenetic changes in germ cells might persist or alter early development in offspring is still unclear. Intriguingly, interindividual variation in body mass index (BMI; calculated as weight in kilograms divided by height in meters squared) in humans was recently reported to be associated with methylation at a putative metastable epiallele in the pro-opiomelanocortin (*POMC*) gene, and analysis of family trios indicated that paternal somatic DNA methylation correlated strongly with DNA methylation patterns in offspring, whereas maternal methylation did not.^16^ Moreover, in the Newborn Epigenetics Study (NEST) cohort, paternal BMI greater than 30 was associated with reduced DNA methylation at several imprinted loci in umbilical cord leukocytes,^17^ but maternal vs paternal obesity had differential associations with DNA methylation of *IGF2*.^18^ However, few studies have examined the effects of paternal obesity on offspring epigenetic marks on a genome-wide basis, or the degree to which such marks may be associated with childhood weight status.

We investigated the independent associations of paternal and maternal BMI with the offspring epigenome with the goal of identifying individual genomic loci and genomic features associated with paternal obesity. For those genomic regions that were differentially methylated in newborns relative to paternal BMI, we examined the extent to which DNA methylation patterns were retained in later life and associated with childhood weight status. We report that paternal periconceptional BMI is significantly associated with cord blood DNA methylation in newborns. For some loci, this association persists in early childhood leukocytes at ages 3 years and 7 years, highlighting an important association of paternal obesity with the childhood epigenome and metabolic risk.

## Methods

### Human Participants

We analyzed participants in Project Viva, a US longitudinal prospective birth cohort study of mothers and children. Detailed protocols, study design, and methods used for Project Viva have been previously published.^19^ Briefly, pregnant women were recruited during their first prenatal visit from obstetric offices of Atrius Harvard Vanguard Medical Associates in eastern Massachusetts between April 1999 and July 2002; the current analyses were done between July 2017 and October 2019. Maternal prepregnancy BMI was calculated from self-reported height and prepregnancy weight, assessed at the first prenatal visit (median 9.9 weeks’ gestation). At each visit, research assistants measured weight with a digital scale and child’s height using a calibrated stadiometer. We used data from the Centers for Disease Control and Prevention 2000 growth charts^20^ to calculate weight-for-age *z* scores and BMI percentiles.

Assessment of paternal BMI was based on maternal report of paternal weight and height at study enrollment. Of the total 2128 mother-infant pairs in the cohort, cord blood DNA methylation data were available for 485 infants, and BMI was available for 470 fathers. We excluded infants with gestational age less than 34 weeks and infants born to mothers with gestational, type 1, or type 2 diabetes or preeclampsia. Thus, a total of 429 father-mother-infant triads were available for analysis. Of these, 107 had blood methylation data available in early childhood (median [range] age, 3.3 [2.9-4.9] years) and 400 had blood methylation data available during midchildhood (median [range] age, 7.7 [6.7-10.5] years). Cord blood samples were collected between 1999 and 2003, early childhood samples between 2003 and 2006, and midchildhood samples between 2007 and 2010.

We evaluated persistence of cord blood associations in children with available blood methylation measurements in early childhood and midchildhood with complete exposure and covariate information. Participant flow is shown in eTable 1 in the Supplement. Written informed consent was obtained from the mother at each study visit and verbal assent from children at the midchildhood visit. The Harvard Pilgrim Health Care institutional review board approved all protocols and procedures. Reporting of this study follows the Strengthening the Reporting of Observational Studies in Epidemiology (STROBE) reporting guideline.

### Blood Sampling for DNA Methylation

Trained medical personnel obtained umbilical cord blood at delivery; additional nonfasted blood samples were obtained during early childhood and midchildhood study visits. Cord blood samples were stored in a dedicated refrigerator at 4 °C and transported to a central location within 24 hours of sample collection. Similarly, whole blood samples collected during early childhood and midchildhood were stored at 4 °C and transported to the central storage location for sample processing; trained laboratory staff processed the samples on the day of arrival. Whole blood samples were centrifuged to separate the buffy coat from plasma and red blood cells, and the buffy coat was transferred into a red blood cell lysis solution. The solution was then centrifuged to obtain the white blood cell pellet. Aliquots were stored at −80 °C until analysis.

### Analysis of DNA Methylation

Whole blood DNA was extracted using the Puregene Kit (Qiagen) and bisulfite converted using the EZ DNA Methylation-Gold Kit (Zymo Research). Samples were randomly allocated to chips and plates and analyzed using Infinium Human Methylation 450 BeadChip (Illumina Inc), which interrogates more than 485 000 CpG sites simultaneously at a single-nucleotide resolution, covering 99% of RefSeq genes; assays were performed in 2014.

### Statistical Analysis

All hypothesis tests were 2-sided. We specified an a priori significance threshold of *P* < .05 after correcting for multiple comparisons using false discovery rates and Bonferroni correction. We adjusted for maternal BMI in our primary analyses (model 1). In parallel, to test whether the association between paternal BMI and offspring epigenetic marks may be influenced by maternal obesity, we also analyzed the association between paternal BMI and offspring epigenetic marks stratified according to maternal BMI categories (secondary analyses, models 2A and 2B). We additionally adjusted for maternal age, gestational weight gain, household income, maternal education, maternal smoking, maternal alcohol use, marital status, infant’s sex, race/ethnicity, gestational age at delivery, mode of delivery, birth weight, batch effects, and estimated nucleated cell types (percentage of CD8^+^ lymphocytes, CD4^+^ lymphocytes, natural killer cells, monocytes, B-cells, granulocytes, and nucleated red blood cells) in all analyses. Statistical procedures for quality control and epigenome-wide analysis of DNA methylation are included as eMethods in the Supplement. Q-Q plots and *P* value distributions are shown in eFigure 1 in the Supplement. We used the Bioconductor package in R software version 3.4.1 (R Project for Statistical Computing) to perform all bioinformatics processing and statistical analyses.

## Results

### Clinical Characteristics

A total of 429 father-mother-child triads had complete data, including paternal and maternal BMI and cord blood DNA methylation; 107 and 400 participants had DNA methylation data from blood samples available for analysis from early childhood (2.9-4.9 years) and midchildhood (6.7-10.5 years), respectively. Demographic information is summarized in Table 1. Mean (SD) periconception paternal BMI was 26.4 (4.0) and mean maternal prepregnancy BMI was 24.5 (5.2); 268 fathers had BMI greater than or equal to 25 (mean [SD], 28.5 [3.3]) and 161 had BMI less than 25 (mean [SD], 22.8 [1.8]). Infants born to fathers with BMI greater than or equal to 25 had higher mean (SD) birth weight (3610 [480] g vs 3502 [508] g; *P* = .03) and mean (SD) birth weight–for–gestational age *z* score (0.38 [0.91] vs 0.11 [0.96] *P* = .004) relative to infants of fathers with BMI less than 25. We noted a significant positive correlation (*r* = 0.32) between paternal and maternal BMI, and maternal BMI differed significantly between the group with paternal BMI greater than or equal to 25 and the group with BMI less than 25 (mean [SD] maternal BMI, 25.1 [5.2] vs 23.4 [5.0]; *P* < .001). Positive correlations between BMI of both parents were also observed after dichotomizing the study population by maternal BMI less than 25 vs greater than or equal to 25 and, similarly, in the subsets with paternal BMI less than 25 vs greater than or equal to 25. Sample sizes according to maternal and paternal BMI less than 25 and 25 or greater are presented in eTable 2 in the Supplement. 

**Table 1.** Participant Demographic and Clinical Characteristics According to Paternal BMI Category

Characteristic	No. (%)			*P* Value^a^
	Overall (N = 429)	Paternal BMI <25 (n = 161)	Paternal BMI ≥25 (n = 268)	
Parental				
BMI, mean (SD)				
Paternal	26.4 (4.0)	22.8 (1.8)	28.5 (3.3)	<.001
Maternal	24.5 (5.2)	23.4 (5.0)	25.1 (5.2)	<.001
Maternal BMI				
<25	282 (66)	122 (76)	160 (60)	<.001
≥25	147 (34)	39 (24)	108 (40)	
Maternal age at enrollment, mean (SD), y	32.1 (5.3)	31.9 (5.4)	32.2 (5.2)	.58
Maternal smoking status				
Never	293 (68)	116 (72)	177 (66)	.08
Former	93 (22)	26 (16)	67 (25)	
Smoked during pregnancy	43 (10)	19 (12)	24 (9)	
Mother graduated college	284 (66)	116 (72)	168 (63)	.05
Household income >$70 000/y	157 (40)	55 (37)	102 (41)	.41
Infant				
Female	206 (48)	70 (43)	136 (51)	.14
Race/ethnicity				
Black	48 (11)	20 (12)	28 (10)	.31
Hispanic	25 (6)	6 (4)	19 (7)	
Asian	14 (3)	8 (5)	6 (2)	
White	293 (68)	110 (68)	183 (68)	
Other	49 (11)	17 (11)	32 (12)	
Birth weight, mean (SD), g	3569 (493)	3502 (508)	3610 (480)	.03
Gestational age, mean (SD), wk	39.8 (1.4)	39.8 (1.3)	39.8 (1.4)	.99
Birth weight–for–gestational age *z* score, mean (SD)	0.28 (0.94)	0.11 (0.96)	0.38 (0.91)	.004
Mode of delivery				
Vaginal	360 (84)	142 (88)	218 (81)	.06
Cesarean	69 (16)	19 (12)	50 (19)	

Abbreviation: BMI, body mass index (calculated as weight in kilograms divided by height in meters squared).

^a^ *P* values refer to χ^2^ test comparing paternal BMI less than 25 vs greater than or equal to 25 for the entire cohort.

### CpG Methylation Analysis

#### Associations Between Paternal BMI and Cord Blood DNA Methylation

**Figure 1.**
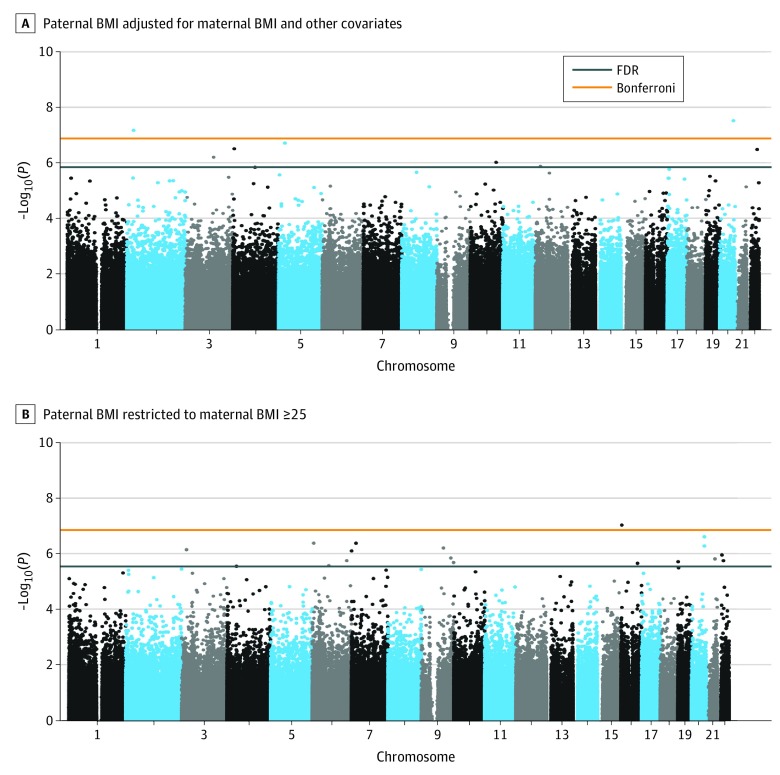
Epigenome-wide DNA Methylation Analyses for Association of Paternal Body Mass Index (BMI) With Cord Blood DNA Methylation A, The exposure for model 1 was paternal BMI (calculated as weight in kilograms divided by height in meters squared) adjusted for maternal prepregnancy BMI and other covariates. B, The exposure for model 2 was paternal BMI restricted to mothers with prepregnancy BMI 25 or greater. The x-axis shows the chromosomal location, while the y-axis shows the negative log_10_ *P* value for the association between paternal BMI and methylation (M-value) at a given CpG locus (ie, greater −log_10_(*P*) indicates greater strength of association). The orange line indicates Bonferroni-adjusted genome-wide significance; blue line, false discovery rate (FDR) *q* less than .05.

**Figure 2.**
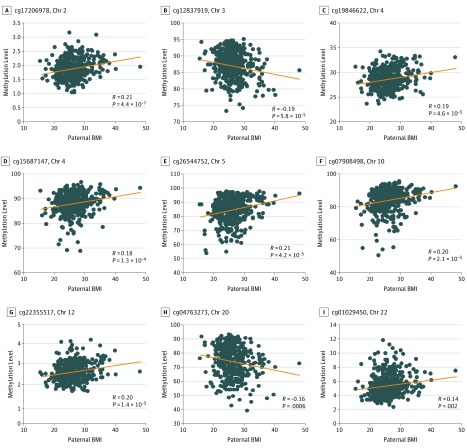
Association of Paternal Periconception Body Mass Index (BMI) and Cord Blood DNA Methylation for 9 Top-Ranking CpG Sites The x-axis shows paternal BMI (calculated as weight in kilograms divided by height in meters squared) and the y-axis shows DNA methylation (M-value). Results are adjusted for maternal prepregnancy BMI, maternal age, gestational weight gain, household income, maternal education, maternal smoking, maternal alcohol use, marital status, infant’s sex, race/ethnicity, gestational age at delivery, mode of delivery, birth weight, batch effects, and estimated nucleated cell types from cord blood (percentage of CD8^+^ lymphocytes, CD4^+^ lymphocytes, natural killer cells, monocytes, B-cells, granulocytes, and nucleated red blood cells). Results are shown for cg17206978 near *CENPA *(A), cg12837919 near *LSAMP* (B), cg19846622 near *MSX1* (C), cg15687147 near *FAM190A* (D), cg26544752 near *CDH10* (E), cg07908498 near *SORCS3* (F), cg22355517 near *PDE3A* (G), cg04763273 near *TFAP2C* (H), and cg01029450 near *ARFGAP3* (I). All CpG loci had false discovery rate *q* less than .05.

We performed a CpG-by-CpG analysis to examine the association of paternal BMI (exposure) and DNA methylation in cord blood (outcome). Because of the correlation between paternal BMI and maternal BMI, we adjusted for maternal BMI as a continuous variable in our primary analyses (model 1). In adjusted linear regression models, DNA methylation at 2 sites (cg04763273 and cg17206978) was significantly associated with paternal BMI after Bonferroni adjustment (Figure 1A). DNA methylation at 9 CpG sites was associated with paternal BMI, with *q* less than .05 (Figure 2). DNA methylation at cg04763273 on chromosome 20 decreased by 5% for every 1-unit increase in paternal BMI (*P* = 3.13 × 10^−8^). This CpG is located in an uncharacterized complementary DNA (cDNA) generated from human testis (*AK097528*); the nearest known gene is transcription factor AP-2 gamma (*TFAP2C*, 295443 base pair [bp] distance). Moreover, methylation at cg17206978 was also associated with paternal BMI (methylation increased by 1% for every 1-unit increase in paternal BMI; *P* = 6.95 × 10^−8^); this CpG is located in a CpG island within the 200-bp upstream promoter region of centromere protein A (*CENPA*) on chromosome 2. Gene annotations and effect sizes for the 9 top-ranking CpG sites are shown in Table 2.

**Table 2.** Genomic Loci at Which Paternal BMI Is Associated With DNA Methylation in Offspring Blood^a^

CpG	Chr	Nearest Gene	Distance to TSS, Base Pairs	Genomic Location	Relation to CpG Island	Gene Name	Cord Blood		Age 3 y		Age 7 y	
							Difference in DNA Methylation^b^	*P* Value	Difference in DNA Methylation	*P* Value	Difference in DNA Methylation	*P* Value
**Adjusted for Maternal BMI**^c^												
cg17206978	2	*CENPA*	0	TSS200	Island	Centromere protein A	0.01	7.0 × 10^−8^^d^	−0.00005	.66	0.0004	.96
cg12837919	3	*LSAMP*	645 389	Intron	OpenSea	Tumor suppressor candidate 7 (non–protein coding)	−0.02	6.4 × 10^−7^	−0.002	.11	1.90 × 10^−6^	.96
cg15687147	4	*FAM190A*	0	Body	OpenSea	Coiled-coil serine rich protein 1	0.03	1.5 × 10^−7^	0.002	.12	0.0001	.85
cg19846622	4	*MSX1*	−432	TSS1500	Island	Msh homeobox 1	0.008	3.2 × 10^−7^	−0.0003	.69	5.00 × 10^−7^	.99
cg26544752	5	*CDH10*	119 235	Body	OpenSea	Cadherin 10	0.05	2.0 × 10^−7^	0.004	.04	−0.0007	.23
cg07908498	10	*SORCS3*	24 101	Body	OpenSea	Sortilin related VPS10 domain containing receptor 3	0.03	9.8 × 10^−7^	0.004	.01	0.0002	.47
cg22355517	12	*PDE3A*	0	First exon	Island	Phosphodiesterase 3A	0.01	1.4 × 10^−6^	0.0003	.14	0.0001	.35
cg04763273	20	*TFAP2C*	295 443	Intron	S_Shore	Transcription factor AP-2 gamma	−0.05	3.1 × 10^−8^^d^	−0.008	.0002^d^	−0.003	.004^d^
cg01029450	22	*ARFGAP3*	0	TSS200	Island	ADP ribosylation factor GTPase activating protein 3	0.02	3.4 × 10^−7^	0.0001	.80	0.0006	.15
**Subset With Maternal BMI ≥25**^e^												
cg08524210	3	*VENTXP7*	558 683	NA	NA	VENT homeobox pseudogene 7	−0.04	6.8 × 10^−7^	−0.004	.10	−0.03	.31
cg07312445	4	*NSUN7*	0	5′UTR; first exon	Island	NOP2/Sun RNA methyltransferase family member 7	0.02	2.7 × 10^−6^	0.02	.32	0.01	.40
cg00213729	6	*B3GAT2*	−241	TSS1500	S Shore	Beta-1,3-glucuronyltransferase 2	−0.03	2.5 × 10^−6^	−0.03	.51	0.05	.81
cg18712083	6	*NRN1*	−48 481	Intron	N Shore	Neuritin 1	−0.04	4.0 × 10^−7^	−0.001	.91	0.002	.54
cg23130766	6	*KATNA1*	0	Intron	Island	Katanin catalytic subunit A1	0.02	1.6 × 10^−6^	0.002	.93	0.72	.51
cg11241627	7	*FERD3L*	0	TSS200	S Shore	Fer3 like bhlh transcription factor	0.02	4.0 × 10^−7^	−0.01	.22	0.01	.77
cg13872065	7	*FAM20C*	−265	TSS1500	N Shore	FAM20C, Golgi associated secretory pathway kinase	0.02	7.5 × 10^−7^	0.31	.009	0.09	.23
cg00785831	9	*ABCA2*	−1612	Body	N Shore	ATP binding cassette subfamily A member 2	−0.05	1.9 × 10^−6^	−0.005	.94	−0.008	.68
cg14320496	9	*RABEPK*	0	NA	N Shelf	Rab9 effector protein with kelch motifs	−0.04	1.3 × 10^−6^	−0.02	.44	−0.007	.58
cg21925493	9	*CARD19*	0	Body	Island	Caspase recruitment domain family member 19	0.01	5.9 × 10^−7^	0.01	.19	−0.006	.62
cg07451886	16	*MMP25*	0	Body	NA	Matrix metallopeptidase 25	0.02	8.9 × 10^−8^^d^	0.02	.32	0.001	.21
cg08862162	16	*TAT*	0	5′UTR	NA	Tyrosine aminotransferase	−0.04	2.1 × 10^−6^	−0.06	.03	0.001	.65
cg02534744	19	*CFD* or *ADIPSIN*	2561	Body	Island	Complement factor D	0.03	1.8 × 10^−6^	0.01	.80	0.03	.86
cg04763273	20	*TFAP2C*	296 425	Intron	S Shore	Transcription factor AP-2 gamma	−0.07	2.3 × 10^−7^	−0.11	.007	−0.05	.05
cg11868041	20	*TFAP2C*	295 443	Intron	S Shelf	Transcription factor AP-2 gamma	−0.02	5.0 × 10^−7^	0.003	.91	−0.07	.45
cg24459147	21	*SIM2*	−2410	NA	Island	Single-minded family bhlh transcription factor 2	0.02	1.4 × 10^−6^	−0.01	.21	0.03	.66
cg16692439	22	*ASPHD2*	0	Body	S Shore	Aspartate beta-hydroxylase domain containing 2	−0.02	1.7 × 10^−6^	−0.02	.22	0.08	.84
cg16880392	22	*PI4KAP1*	67	Intron	N Shelf	Phosphatidylinositol 4-kinase alpha pseudogene 1	−0.03	1.0 × 10^−6^	−0.02	.40	0.02	.23

Abbreviations: BMI, body mass index (calculated as weight in kilograms divided by height in meters squared); Chr, chromosome; NA, not applicable; TSS, transcriptional start site.

^a^ Paternal BMI (exposure) was modeled as a continuous variable, with child’s DNA methylation (β-coefficient) in cord blood or peripheral blood (age 3 years and age 7 years) as the outcome. All associations are adjusted for maternal age, education, smoking, mode of delivery, child race/ethnicity, gestational age, sex, and cell type proportions.

^b^ Difference in DNA methylation is the relative increase or decrease in DNA methylation (β-coefficient) with a 1-point increase in preconception paternal BMI (eg, 0.01 difference in DNAm is equivalent to 1% increase in methylation).

^c^ Top-ranking genetic loci associated with paternal BMI independent of maternal BMI. These 9 loci were identified in the fully adjusted analyses in cord blood (n = 429), where maternal prepregnancy BMI was included as a covariate. Persistence of associations is shown for age 3 (n = 110) and age 7 (n = 236).

^d^ Significant *P* value after Bonferroni correction.

^e^ Top-ranking genetic loci where paternal BMI is associated with offspring DNA methylation, in subset with maternal BMI greater than or equal to 25 (n = 108). No CpG loci were significantly altered by paternal BMI in the subset with maternal BMI less than 25 (not shown). Persistence of associations is shown for age 3 years and age 7 years. Table includes only loci with *q* less than .05 in cord blood analysis.

#### Association Between Paternal BMI and Cord Blood DNA Methylation Stratified by Maternal Obesity

We next stratified the population according to maternal overweight and obesity status (BMI <25 vs ≥25, model 2A and 2B, respectively) to assess contributions of paternal and maternal obesity on the offspring epigenome. Strikingly, no CpG loci were differentially methylated in association with paternal BMI in the subset of mothers with BMI less than 25 (282 participants; model 2A). By contrast, among mothers with BMI 25 or greater (147 participants; model 2B), methylation at 18 CpG sites showed a significant association between paternal BMI and locus-specific methylation in covariate-adjusted linear models. This suggests that maternal and paternal obesity may act synergistically to influence DNA methylation in the offspring. Among infants born to mothers with BMI 25 or greater, 1 CpG (cg07451886) annotated to the *MMP25* gene was significantly associated with paternal BMI at a Bonferroni-adjusted genome-wide level of significance (β = 0.02; *P* = 8.9 × 10^−8^; Figure 1B). An additional 17 CpG were significant at *q* less than .05 (Table 2). Of note, 2 of the significantly differentially methylated loci, cg04763273 and cg11868041, are located in close proximity to one another in a CpG island within *AK097528*, an uncharacterized cDNA first generated from human testis, and located between *TFAP2C* and bone morphogenetic protein 7 (*BMP7*) (eFigure 2 in the Supplement). As noted earlier, methylation at cg04763273 was also top ranking in the unstratified analysis (model 1).

#### Analysis of Functional Annotations of CpG Sites Associated With Paternal BMI

We next examined reported functional annotations for genes closest to the CpG associated with paternal BMI. Patterns of tissue expression for the genes nearest the differentially methylated CpG are presented in eTable 3 in the Supplement. Of note, several of the top-ranking CpG mapped to genes with wide tissue expression, including in male reproductive tissues (eg, prostate, testis).

We next used motifDB to identify transcription factor binding sites in the 2000-bp sequences surrounding each CpG site identified in the primary analyses with matching score of 95% or higher. By comparison with binding sites identified in a background set of 10 000 randomly selected CpG sites from the DNA methylation array, we identified 133 transcription factor binding sites as enriched (false discovery rate *q* < .05; eTable 4 in the Supplement). Top-ranking binding sites with at least 2 sites included the transcription factors POU2F1, POU2F2, HOXA5, and OLIG1. For the maternal BMI 25 or greater model, 258 transcription factor binding sites were enriched (false discovery rate *q* < .05). These include SP1, SP2, KLF5, and KLF16. Similarly, we performed motif discovery, searching for sequence motifs that are enriched or depleted in the sequences surrounding CpG associated with paternal BMI (eTable 4 in the Supplement). Motif-finding analysis revealed 10 motifs enriched (false discovery rate *q* < .05), including multiple GATA, TFAP2A, ZNF354C, and others. For CpG identified in the maternal BMI 25 or greater model, 10 motifs were enriched at false discovery rate *q* less than .001, including MZF1, MEIS1, ETS1, TFAP2A, and KLF5.

### Persistence Analysis: Association Between Paternal BMI and DNA Methylation During Early Childhood

To determine whether site-specific offspring DNA methylation differences associated with paternal BMI might persist with time, we examined DNA methylation in whole blood during early childhood (age 3 years) and midchildhood (age 7 years) for those CpG identified in the cord blood analysis (Table 2). Three of the 9 CpG loci identified in the overall analysis remained significant in the early childhood assessment, cg04763273 (chromosome 20, near *TFAP2C*), cg26544752 (chromosome 5, near *CDH10*), and cg07908498 (chromosome 10, near *SORCS3*). Association with paternal BMI for cg04763273 persisted in both the early (β = −0.008; *P* = .0002) and midchildhood (β = −0.003; *P* = .004) methylation analyses and survived adjustment for multiple comparisons. In the analysis stratified according to maternal BMI 25 or greater, paternal BMI remained associated with early childhood methylation levels at 3 of 18 CpG sites, cg04763273 (chromosome 20, near *TFAP2C*), cg13872065 (chromosome 7, near *FAM20C*), and cg08862162 (chromosome 16, near *TAT*), and cg04763273 remained differentially methylated at midchildhood. However, none of the early childhood and midchildhood associations in the stratified analysis survived adjustment for multiple comparisons.

### Associations of DNA Methylation at CpG Sites Linked to Paternal BMI and Infant Birth Weight

Given that birth weight was greater in offspring of fathers with BMI 25 or greater, and that infant birth weight is associated with many developmentally programmed offspring phenotypes, we examined associations between infant birth weight and DNA methylation at the 9 individual CpG found to be associated with paternal BMI. Cord blood DNA methylation of cg01029450 in the 2-kilobase (kb) promoter region of the *ARFGAP3* gene was inversely associated with infant birth weight (β = −0.0003; SD = 0.0001; *P* = .03 for birth weight in grams; β = −0.17; SD = 0.08; *P* = .02 for birth weight *z* score for gestational age and sex). Methylation at this CpG was also associated with BMI *z* score at age 3 years, but the directionality of association had reversed (β = 0.186; SD = 0.073; *P* = .01). However, analysis of associations with birth weight did not survive adjustment for multiple comparisons.

## Discussion

This study found that paternal BMI was associated with altered DNA methylation in cord blood nucleated cells with genome-wide significance, even after adjustment for maternal BMI and key confounders. We identified a potential additive association of paternal and maternal obesity with the infant epigenome based on analyses stratified by maternal BMI. DNA methylation at *ARFGAP3* was significantly associated with paternal BMI and with lower infant birth weight, a risk factor for future cardiometabolic disease. Locus cg04763273, localized to an intronic region on chromosome 20 between *TFAP2C* and *BMP7*, was hypomethylated in the newborn period, and remained significantly hypomethylated in early childhood and midchildhood. Together, our data suggest that paternal BMI can have a lasting association with the offspring’s epigenetic landscape in humans. Our data, from a large cohort study, are notable because prior analyses of paternal effects on epigenetic marks have relied on experimental models or have analyzed either a limited number of patients or a few candidate loci. To what extent our observed associations might reflect underlying shared genetic or environmental risk factors is unclear from this cross-sectional analysis.

Our findings of concordance between maternal and paternal BMI contrast with those in the Avon Longitudinal Study of Parents and Children,^21^ which examined associations between parental factors and the cord blood epigenome but found that none of the top-ranking genomic loci associated with maternal BMI were associated with paternal BMI. However, paternal BMI was used as a negative control, with analyses restricted to loci found in the primary maternal BMI analyses, and was not examined as a primary exposure. Our results add to the emerging body of literature showing associations of paternal factors with childhood health outcomes. Associations have been discovered between increasing paternal age at conception and higher risk for neurological disorders such as autism and schizophrenia, birth defects (eg, orofacial clefts), and stillbirth.^22^ Moreover, paternal height is associated with birth weight (positive correlation), while paternal smoking is associated with lower birth weight, increased risk of congenital heart defects and orofacial clefts, and increased risk of childhood cancers.^22^ Associations have been found between both paternal BMI and type 1 diabetes and increased risk of obesity and diabetes in offspring.^23^ From a mechanistic standpoint, these associations may be mediated by shared genetics, nutritional and/or environmental factors, epigenetic signals transmitted by male germ cells, or nongenetic factors such as sperm quality or function. Indeed, impaired sperm parameters have been found to be associated with lower offspring birth weight.^24^ Together, these data raise the provocative hypothesis that interventions to optimize fathers’ health prior to conception might influence sperm function, epigenetic marks, and health outcomes in their progeny; this will be an important area for future studies.

We observed associations between paternal periconception BMI and hypomethylation at CpG 04763273 in offspring cord blood; this hypomethylation remained statistically significant at age 3 years and age 7 years. This CpG site is located in the intron of *AK097528*, a cDNA clone generated from human testis. A second CpG site (cg11868041) located within the same CpG island was also top ranking in the analysis of paternal BMI within the subset with maternal BMI 25 or greater (shown in eFigure 2 in the Supplement). These loci are located between the genes *TFAP2C* and *BMP7*, both of which are important developmental genes. *TFAP2C* is a transcription factor involved in craniofacial and skeletal development and in the maintenance of pluripotency; high levels of expression have been reported in mammary and germ cell tumors.^25,26^ *BMP7* encodes a secreted ligand in the transforming growth factor–β family and plays an important role in brown adipose tissue, bone, and kidney development; it may also influence appetite regulation.^27,28^

We found that paternal BMI was associated with increased DNA methylation near the transcription start site for *ARFGAP3*, and methylation at this locus in turn was associated with lower birth weight, lower weight–for–gestational age *z* score, and higher BMI *z* score at age 3 years. The *ARFGAP3* gene encodes a GTPase-activating protein involved in Golgi apparatus function and vesicle trafficking^29^ whose activity is sensitive to phospholipids.^30^ *ARFGAP3* is expressed in the prostate and testis and is responsive to androgens.^31^ Interestingly, several of the other top-ranking loci that were differentially methylated in association with paternal BMI were near genes with high levels of expression in the testis, including cadherin 10 (*CDH10*), sortilin-related VPS10 domain containing receptor 3 (*SORC3*), coiled-coil serine rich protein 1 (*CCSER1* or *FAM190A*), caspase recruitment domain family member 19 (*CARD19* or *C9ORF89*), and NOP2/Sun RNA methyltransferase family member 7 (*NSUN7*). Moreover, *TFAP2C* has been proposed as a marker for undifferentiated male germ cells.^32^ Of note, *NSUN7* has been associated with sperm motility and fertility in mice and humans.^33,34^ Many of the differentially methylated loci identified in our analysis were near genes with high levels of expression in brain (eg, *ABCA2*, *ASPHD2*, *B3GAT2*, *CDH10*, and *SORCS3*), which is intriguing given that studies of the sperm epigenome before and after endurance training in men have shown that exercise-induced epigenetic remodeling occurs at loci linked to nervous system development and function,^35^ while weight loss surgery alters sperm epigenetic marks at loci linked to neural control of appetite.^14^

Another top-ranking differentially methylated locus was centromere protein A (*CENPA*), which encodes a centromere protein with a histone H3–related domain. This pattern is interesting given that cell cycle pathways appear to be sensitive to prenatal perturbations, as altered expression of cell cycle genes has been reported in multiple models of prenatal perturbation, including intrauterine growth restriction, maternal obesity, and paternal high-fat diet.^7,8,36,37,38,39^

Epigenetic marks in sperm differ from those in other tissues in several important ways. For example, chromatin architecture is remodeled during spermatogenesis, with the majority (90%-95%) of histones replaced with protamine residues to allow greater compaction of the nucleus for optimal motility.^40^ Interestingly, paternal high-fat diet may influence the extent to which histones are retained in the sperm nucleosome in mice^41^; it is not known whether this might also be true in humans. After fertilization, DNA methylation marks are largely erased from the sperm genome and decrease further during a second wave of demethylation arising during gametogenesis in the early embryo.^42^ However, some genomic sites in sperm escape methylation erasure, providing an avenue for paternal intergenerational transmission of phenotypes. For example, demethylation is near complete within imprinting control regions and CpG-dense areas, whereas transposons and intergenic regions are more likely to remain methylated.^43^ Consistent with this possibility, several of the differentially methylated loci in our analysis were located in intergenic regions. Our findings are consistent with studies in rodents showing that DNA methylation marks in the male germ cell may be influenced by ancestral exposure to undernutrition, diabetes, and other stressors.^4,6,9^

### Strengths and Limitations

Some of the strengths of our study include the large sample size, the detailed longitudinal assessments throughout pregnancy and childhood, the use of epigenome-wide DNA methylation as outcome (rather than selected candidate genes), adjustment for numerous biological and socioeconomic confounders and covariates, and the analysis for persistence of methylation patterns from birth to age 7 years.

We also acknowledge some limitations to our study. First, paternal BMI was ascertained based on mother’s report of the father’s height and weight and was not directly measured. Self-reported height and weight may be biased by social desirability and other factors,^44^ but it is unclear whether this would also be the case when an individual is reporting on their partner’s measurements. Many other studies examining effects of paternal BMI have similarly relied on mother’s report of father’s height and weight.^17,45,46^ Another limitation is that we cannot rule out an effect of genetic polymorphisms on DNA methylation patterns. However, we did exclude from analysis all CpG probes located within 1 bp of known single-nucleotide polymorphisms. It is also reassuring that the scatterplots for the associations between paternal BMI and DNA methylation at individual sites were not bimodal, as might be expected if the differences were due to differences in single-nucleotide polymorphism frequencies. A third limitation is that we were unable to determine the mechanisms by which paternal BMI may influence epigenome-wide methylation patterns in progeny. From a mechanistic standpoint, paternal obesity might influence DNA methylation patterns in infant cells via a signal transmitted in sperm, but we did not have male germ cells available for analysis. Fourth, given the observed strong correlation between the BMIs of mothers and fathers, as well as potential for clustering of dietary and lifestyle risk factors for obesity within the family units, we cannot exclude potential effects of shared influences such as genetics, diet, lifestyle, or environmental exposures that might simultaneously affect paternal BMI, child BMI, and DNA methylation. These potential interactions are depicted in eFigure 3 in the Supplement.

## Conclusions

This cohort study found that paternal periconception BMI was significantly associated with epigenome-wide DNA methylation patterns in offspring at birth. We further observed that associations between paternal BMI and DNA methylation in a subset of loci remained detectable at age 3 years and age 7 years, suggesting that paternal obesity may impart a lasting epigenetic legacy. Paternal BMI was also associated with infant birth weight, a risk factor for future cardiometabolic disease. These data suggest that paternal obesity should be viewed as a risk factor for childhood health and disease outcomes.

## Notes

Supplement. **eMethods.** DNA Methylation and Statistical Analysis **eFigure 1.** Q-Q Plot and *P*-value Distribution for CpG-by-CpG Analysis in Cord Blood **eFigure 2.** Associations of Paternal BMI and DNA Methylation Surrounding Top-Ranking CpG cg04763273 on Chromosome 20 **eFigure 3.** Schematic Summary of the Effect of Paternal BMI on DNA Methylation Patterns and Childhood BMI **eTable 1.** Sample Sizes at the Newborn, Early Childhood (3 y), and Mid-childhood (7 y) Visits, After Exclusions **eTable 2.** Cord Blood DNA Methylation Sample Size According to Parental BMI Category **eTable 3.** Tissue Expression Patterns for Genes Near Differentially Methylated Loci Identified in Model 1 and Model 2b **eTable 4.** Motif Enrichment and Transcription-Factor Binding Analysis **eReferences.**
