# Supplementary material for: Association of Periconception Paternal Body Mass Index With Persistent Changes in DNA Methylation of Offspring in Childhood
Source: JAMA Netw Open. 2019 Dec 27;2(12):e1916777. doi: 10.1001/jamanetworkopen.2019.16777 (PMC6991200; doi:10.1001/jamanetworkopen.2019.16777)
Supplement: Supplement. — eMethods. DNA Methylation and Statistical Analysis eFigure 1. Q-Q Plot and P-value Distribution for CpG-by-CpG Analysis in Cord Blood eFigure 2. Associations of Paternal BMI and DNA Methylation Surrounding Top-Ranking CpG cg04763273 on Chromosome 20 eFigure 3. Schematic Summary of the Effect of Paternal BMI on DNA Methylation Patterns and Childhood BMI eTable 1. Sample Sizes at the Newborn, Early Childhood (3 y), and Mid-childhood (7 y) Visits, After Exclusions eTable 2. Cord Blood DNA Methylation Sample Size According to Parental BMI Category eTable 3. Tissue Expression Patterns for Genes Near Differentially Methylated Loci Identified in Model 1 and Model 2b eTable 4. Motif Enrichment and Transcription-Factor Binding Analysis eReferences. [file jamanetwopen-2-e1916777-s001.pdf]

Supplementary Online Content

Noor N, Cardenas A, Rifas-Shiman SL, et al. Association of periconception paternal body mass index with persistent changes in DNA methylation of offspring in childhood. *JAMA Netw Open*. 2019;2(12):e1916777. doi:10.1001/jamanetworkopen.2019.16777

eMethods. DNA Methylation and Statistical Analysis
eFigure 1. Q-Q Plot and *P*-value Distribution for CpG-by-CpG Analysis in Cord Blood
eFigure 2. Associations of Paternal BMI and DNA Methylation Surrounding Top-Ranking CpG cg04763273 on Chromosome 20
eFigure 3. Schematic Summary of the Effect of Paternal BMI on DNA Methylation Patterns and Childhood BMI
eTable 1. Sample Sizes at the Newborn, Early Childhood (3 y), and Mid-childhood (7 y) Visits, After Exclusions
eTable 2. Cord Blood DNA Methylation Sample Size According to Parental BMI Category
eTable 3. Tissue Expression Patterns for Genes Near Differentially Methylated Loci Identified in Model 1 and Model 2b
eTable 4. Motif Enrichment and Transcription-Factor Binding Analysis
eReferences.

This supplementary material has been provided by the authors to give readers additional information about their work.

© 2019 Noor N et al. *JAMA Network Open.*

eMethods: DNA Methylation and Statistical Analysis

DNA Methylation Analysis and Quality Control. Extracted DNA samples from cord blood and venous blood (for age 3 and age 7 visits) were shipped to Illumina Inc., and analyzed using the Infinium HumanMethylation450 BeadChip (Illumina, San Diego, CA) following standard manufacturer's protocols. The HumanMethylation450 BeadChip measures DNA methylation at >485,000 CpG sites simultaneously at a single nucleotide resolution; the chip includes one or more CpG sites for 99% of RefSeq genes.

We carried out pre-processing, filtering and quality control procedures for DNA methylation data as described previously for other analyses using Project Viva DNA methylation datasets.[1-3] The same procedures were followed for cord blood, early childhood, and mid-childhood DNA methylation datasets. We processed raw DNA methylation files using the *minfi* package in R from Bioconductor. Samples were excluded as potentially mis-labelled if they were mismatched on sex or genotype (based on rs probes, as described [4]), or deemed to be low in quality. Technical replicates (n=40) were also excluded from the analysis. Correlation coefficients for individual probes among all technical replicates ranged from 0.98 to 1. We excluded individual probes if they had non-significant detection (P>0.05) for more than 1% of the samples. Additionally, we excluded non-CpG probes (i.e. rs and ch) and probes in X and Y chromosomes. SNP-associated probes at either the single base extension or within the target region were removed for SNPs that have a minor-allele frequency of >5%. Previously identified non-specific and cross-reactive probes within the array along with polymorphic CpG loci were also excluded from the analysis.[5]

For background correction and dye-bias equalization, we performed the normal-exponential out-of-band (noob) correction method.[6] Finally, a β-mixture quantile intra sample normalization procedure (BMIQ) was applied to the resulting data to reduce the potential bias that can arise from type 2 probes[5]. For each CpG site, methylation is reported as average β-value = $M/(M + U + \varepsilon)$, where M and U represent the average fluorescence intensity from the

© 2019 Noor N et al. *JAMA Network Open.*

probe corresponding to the methylated and unmethylated target CpG and $\varepsilon = 100$ is a small quantity to protect against division by zero. Thus, the average β-value is an interval scaled quantity between zero and one interpreted as the fraction of DNA molecules whose target CpG is methylated.

We used ComBat[7] to correct for batch effects from plate and other potential sources of technical variability in methylation measurements, including paternal BMI as the variable of interest. We visually inspected the effectiveness of adjustment for batch using the four main principal components before and after batch adjustment. Strip plots of control probes were visually examined for bisulfite conversion and specificity. Density plots for the β-values were examined across samples at each normalization step. Methylation values on the β-scale were logit transformed to M-values, as previously described, for differential analysis of DNA methylation.[8] All tables and results are presented on the β-value scale.

Statistical Analysis of Clinical Data. Sample size and percentage or means and standard deviations (SD) for all covariates were calculated to describe characteristics of infants born to normal weight fathers (BMI <25 kg/m^2) versus overweight or obese fathers (BMI $\geq$25 kg/m^2).

Statistical Analysis of CpG Methylation. We performed a genome-wide CpG by CpG analysis of DNA methylation in cord blood, using robust linear regression models with heteroskedasticity-consistent estimators to model DNA methylation of each individual CpG on the M-value scale as the dependent variable and paternal BMI exposure (continuous variable) as the main predictor while adjusting for covariates. Adjustment covariates were selected a priori, including maternal age, maternal pre-pregnancy BMI, gestational weight gain, household income, maternal education, maternal smoking, maternal alcohol use, marital status, infant's sex, child race/ethnicity (as reported by mothers), gestational age at delivery, mode of delivery, birth weight, batch effects, and estimated nucleated cell types from cord blood (percentage of CD8+,

© 2019 Noor N et al. *JAMA Network Open.*

CD4+, Natural Killer cells, monocytes, B-cells, granulocytes, and nucleated red blood cells) (Model 1). Cell type proportions in cord blood were estimated from DNA methylation data using a reference panel of nucleated cells isolated from cord blood (leukocytes and nucleated red blood cells).[9,10] An adult leukocyte reference panel was used to estimate cell type proportions for blood samples collected in early- and mid-childhood as implemented in *minfi* [11] and as described previously.[1-3] In additional analyses to determine whether there is an interaction of maternal and paternal BMI on the offspring methylome, data were stratified by maternal BMI <25 and >=25 kg/m^2 (Model 2a and 2b) and paternal BMI was modeled as continuous variable. After inclusion of cell-type estimation and other adjustment covariates, the genomic inflation factor (λ) for the Epigenome-Wide Analysis was 1.23 for Model 1, 0.98 for Model 2a, and 1.34 for Model 2b, with values close to 1 indicating that results were unlikely driven by population stratification or cryptic relatedness (**eFigure 1**).

Statistical significance for the CpG-by-CpG analysis was corrected for multiple testing by controlling the false discovery rate; we considered associations with FDR *q* value <0.05 as statistically significant. We also report CpGs reaching a Bonferroni-adjusted level of significance (P<1.3 E-07) for cord blood analyses. Model fit and assumptions were examined using scatterplots of the standardized residuals vs. the fitted values, residuals vs. the leverage, and quantile-quantile plots of the standardized residuals. Further, multivariable robust linear regression models were used to examine the association between statistically significant cord blood CpG sites and infant phenotypes of birth weight, and birth weight for gestational age z-score. A P-value of <0.05 was taken as statistically significant in the analysis of DNA methylation versus birth weight only. To evaluate the persistence of association, we utilized multivariate robust linear regression models adjusting for covariates, and considered nominal P< 0.05 as indicating the persistence of epigenetic alterations in early or mid-childhood, as we tested only the individual CpG site that we already had found in cord blood reaching our FDR threshold. We used gene annotations from Illumina HumanMethylation450 v1.2 Manifest File; for sites

© 2019 Noor N et al. *JAMA Network Open.*

where annotations were missing we report the nearest gene symbol. Tissue expression annotations were manually searched in the NCBI Gene database based on an RNA-seq study representing 27 different tissues in 95 human individuals.[12] To evaluate potential regulatory activity of epigenetic associations a transcription factor binding site analysis and motif search were performed using the R packages MotifDb and motifRG, respectively. For transcription factor binding and motif search analyses, 10,000 random sequences of 2000 bp length surrounding CpG sites included on the Illumina HumanMethylation450 platform were used for background. All analyses were carried out using R/Bioconductor.

© 2019 Noor N et al. *JAMA Network Open.*

eFigure 1. Q-Q plot and *P*-value distribution for CpG-by-CpG analysis in cord blood.
e1A. Model 1, paternal BMI (exposure) versus DNA methylation (outcome), fully adjusted model including adjustment for pre-pregnancy maternal BMI.
e1B. Model 2a, paternal BMI (exposure) versus DNA methylation (outcome), stratified by pre-pregnancy maternal BMI < 25 kg/m^2.
e1C. Model 2b, paternal BMI (exposure) versus DNA methylation (outcome), stratified by pre-pregnancy maternal BMI ≥ 25 kg/m^2.
The Q-Q plot is shown on the left, and the P-value histogram is shown on the right.

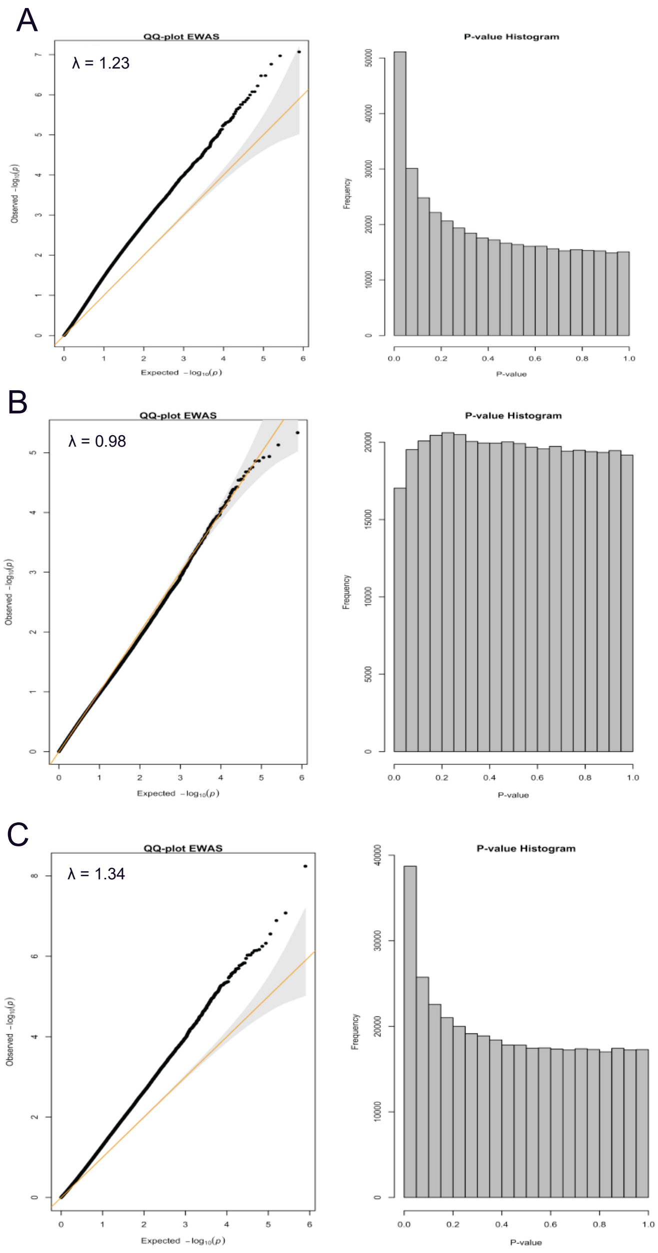

© 2019 Noor N et al. *JAMA Network Open.*

eFigure 2. Associations of paternal BMI and DNA methylation surrounding top-ranking CpG cg04763273 on chromosome 20.

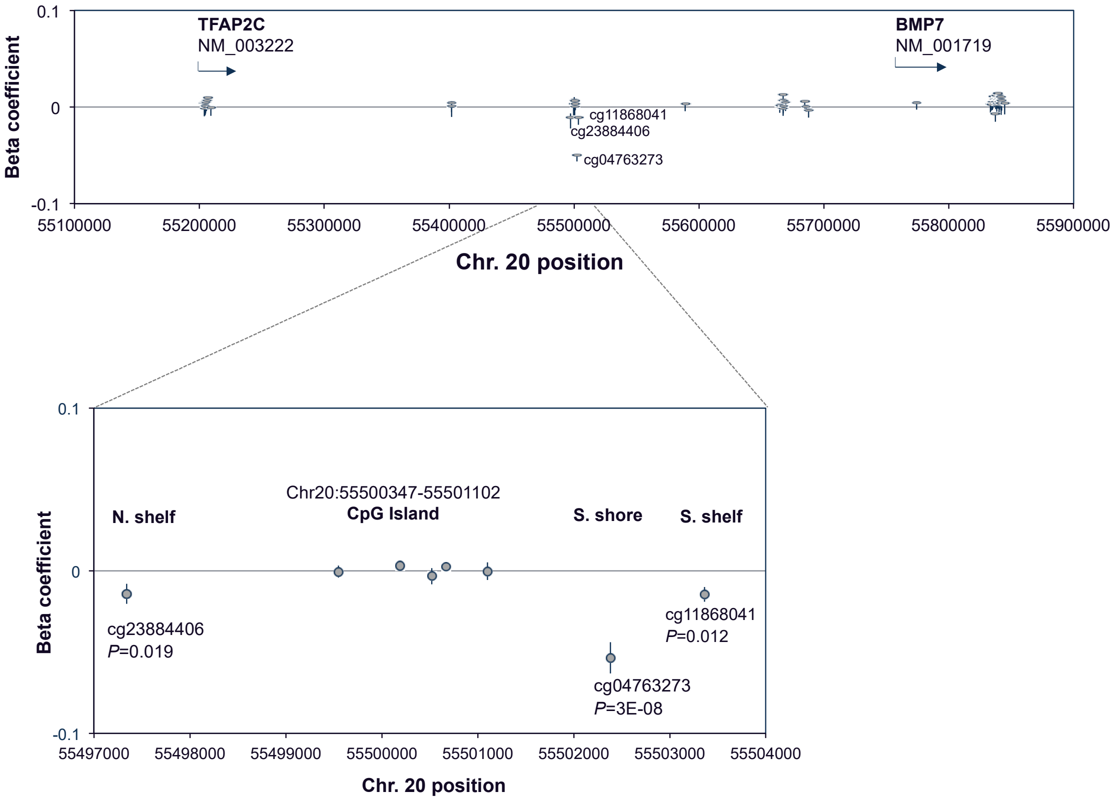

© 2019 Noor N et al. *JAMA Network Open.*

eFigure 3. Schematic summary of the effect of paternal BMI on DNA methylation patterns and childhood BMI.

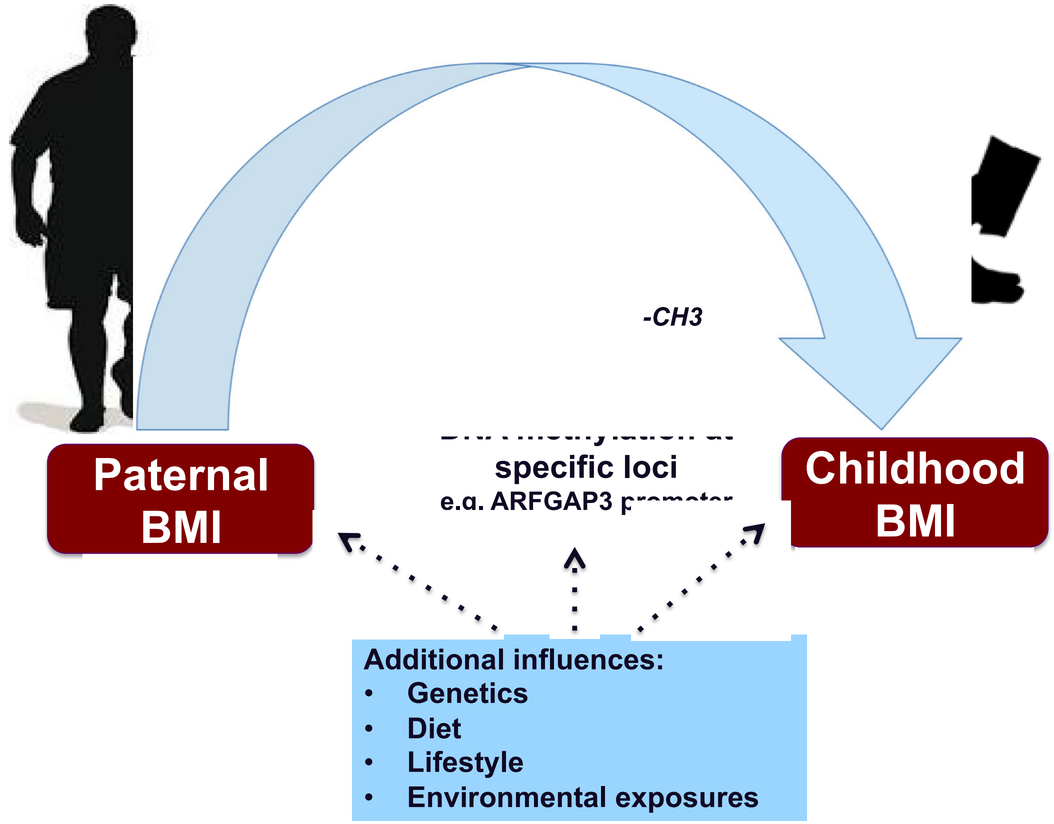

© 2019 Noor N et al. *JAMA Network Open.*

eTable 1. Sample sizes at the newborn, early childhood (3 y), and mid-childhood (7 y) visits, after exclusions. * Covariates not missing: maternal age, maternal BMI, child race/ethnicity, maternal education, maternal smoking, gestational age at delivery, infant sex. Abbreviations, PE: pre-eclampsia, GA: gestational age.

**Participant flow**	**Cord blood**	**3 y**	**7 y**
Valid 450K data	485	120	460
No GDM, no PE, no pre-existing diabetes, GA >=34 wks	444	110	418
Father's BMI not missing	430	108	401
Covariates not missing*	429	107	400

© 2019 Noor N et al. *JAMA Network Open.*

eTable 2. Cord blood DNA methylation sample size according to parental BMI category.

**Maternal pre-pregnancy BMI >=25**	N	%
No	282	65.7
Yes	147	34.3
**Paternal BMI >=25**	N	%
No	161	37.5
Yes	268	62.5
**Maternal BMI >=25 x Paternal BMI >=25**	N	%
No x No	122	28.4
No x Yes	160	37.3
Yes x No	39	9.1
Yes x Yes	108	25.2

© 2019 Noor N et al. *JAMA Network Open.*

eTable 3. Tissue expression patterns for genes near differentially methylated loci identified in Model 1 and Model 2b. Tissue expression based on RNA seq in 27 tissues from 95 individuals, as reported in Fagerberg et al.[12]

**Model 1: Fully adjusted model (effects of paternal BMI, adjusted for maternal BMI).**				
**CpG**	**Chr**	**Gene Symbol**	**Gene Name**	**Tissue expression**
cg17206978	2	CENPA	centromere protein A	Lymph node, appendix, bone marrow, heart
cg12837919	3	LSAMP	limbic system associated membrane protein	Brain, prostate, gall bladder, adrenal
cg19846622	4	MSX1	msh homeobox 1	Endometrium, fat, placenta, brain
cg15687147	4	CCSER1 or FAM190A	coiled-coil serine rich protein 1	Kidney, testis, urinary bladder
cg26544752	5	CDH10	cadherin 10	Brain, prostate, adrenal, testis
cg07908498	10	SORCS3	sortilin related VPS10 domain containing receptor 3	Brain, adrenal, testis
cg22355517	12	PDE3A	phosphodiesterase 3A	Heart, placenta, colon, gall bladder
cg04763273	20	TFAP2C	transcription factor AP-2 gamma	Skin, placenta, esophagus
cg01029450	22	ARFGAP3	ADP ribosylation factor GTPase activating protein 3	Ubiquitous; highest in adrenal, prostate, thyroid, testis
**Model 2b: Subset analysis of effects of paternal BMI among maternal BMI >25 kg/m2**				
**CpG**	**Chr**	**Gene Symbol**	**Gene Name**	**Tissue Expression**
cg08524210	3	VENTXP7	VENT homeobox pseudogene 7	
cg07312445	4	NSUN7	NOP2/Sun RNA methyltransferase family member 7	Testis, thyroid, prostate, kidney
cg18712083	6	NRN1	neuritin 1	Brain, fat, placenta, lung
cg23130766	6	KATNA1	katanin catalytic subunit A1	Testis, ovary, endometrium, lymph node
cg00213729	6	B3GAT2	beta-1,3-glucuronyltransferase 2	Ubiquitous; highest in brain, duodenum, prostate
cg11241627	7	FERD3L	Fer3 like bHLH transcription factor	
cg13872065	7	FAM20C	FAM20C, golgi associated secretory pathway kinase	Ubiquitous; highest in kidney, fat, appendix
cg21925493	9	CARD19	caspase recruitment domain family member 19	Ubiquitous; highest in bone marrow, testis, spleen
cg14320496	9	RABEPK	Rab9 effector protein with kelch motifs	Ubiquitous; highest in liver, testis, brain

© 2019 Noor N et al. *JAMA Network Open.*

cg00785831	9	ABCA2	ATP binding cassette subfamily A member 2	Brain, thyroid, spleen, fat
cg07451886	16	MMP25	matrix metallopeptidase 25	Bone marrow, appendix, spleen, lymph node
cg08862162	16	TAT	tyrosine aminotransferase	Liver
cg02534744	19	CFD or Adipsin	complement factor D	Fat, colon, prostate
cg04763273	20	TFAP2C	transcription factor AP-2 gamma	Skin, placenta, esophagus
cg11868041	20	TFAP2C	transcription factor AP-2 gamma	Skin, placenta, esophagus
cg24459147	21	SIM2	single-minded family bHLH transcription factor 2	Kidney, esophagus, prostate, stomach
cg16880392	22	PI4KAP1	phosphatidylinositol 4-kinase alpha pseudogene 1	Ubiquitous; highest in placenta, brain, testis
cg16692439	22	ASPHD2	aspartate beta-hydroxylase domain containing 2	Brain, stomach, lymph node, skin

© 2019 Noor N et al. *JAMA Network Open.*

eTable 4. Motif enrichment and transcription-factor binding analysis.
e4A. Transcription factor binding sites identified in motif enrichment analysis comparing 1kb upstream and downstream sequences flanking the 9 CpG sites identified in fully adjusted analyses, versus a background set of 10,000 CpG randomly selected from Illumina Human Methylation 450 Beadchip array.
e4B. Transcription factor motifs identified in enrichment analysis comparing 1kb upstream and downstream sequences flanking the 22 CpG sites identified in analyses stratified by maternal BMI >25, versus a background set of 10,000 CpG randomly selected from Illumina Human Methylation 450 Beadchip array.

**e4A. Motif enrichment for CpG in Model 1 (CpG sites associated with paternal BMI, adjusted for maternal BMI.)**				
**Motif**	**Score**	**P-value**	**FDR**	**Transcription factor binding sites**
NNDNACGCTTTCCTNN	6.28	3.30E-10	3.30E-09	jaspar2016-SPI1-MA0080.1 /// jaspar2016-ETS1-MA0098.1
NWGCCCGACCCCWN	5.60	2.17E-08	8.13E-08	jaspar2016-TFAP2A-MA0003.1 /// jaspar2016-KLF5-MA0599.1 /// jolma2013-NR2F1-3
NNWATAAAAAAATBNWNANN	5.58	2.44E-08	8.13E-08	jaspar2016-FOXL1-MA0033.2 /// jaspar2016-SRY-MA0084.1 /// jaspar2016-FOXG1-MA0613.1 /// jaspar2016-FOXD2-MA0847.1 /// jaspar2016-FOXP3-MA0850.1 /// jolma2013-FOXD2-2 /// jolma2013-FOXD3-2 /// jolma2013-FOXJ2-2 /// jolma2013-FOXJ3 /// jolma2013-FOXL1 /// jolma2013-FOXO1 /// jolma2013-FOXP3 /// jolma2013-CDX2 /// jolma2013-HOXC12 /// jolma2013-HOXD12 /// jolma2013-CPEB1
NNAAAAATGCACNN	5.41	6.47E-08	1.62E-07	jaspar2016-ZNF354C-MA0130.1
NNAGAGATGGCANN	5.03	4.82E-07	9.65E-07	jaspar2016-GATA2-MA0036.1 /// jaspar2016-GATA3-MA0037.1 /// jaspar2016-YY1-MA0095.1 /// jaspar2016-NFIC-MA0161.1
NAAAGAAAGCTTNN	4.78	1.72E-06	2.87E-06	
RRAACCACCAATMN	4.51	6.46E-06	6.46E-06	jaspar2016-RUNX1-MA0002.1 /// jaspar2016-ZNF354C-MA0130.1 /// jaspar2016-HOXA5-MA0158.1
RNAAGGGGAGCAGG	4.51	6.46E-06	6.46E-06	jaspar2016-MZF1-MA0056.1 /// jaspar2016-RHOXF1-MA0719.1 /// jolma2013-RHOXF1-2
NYACATCCATCANN	4.51	6.46E-06	6.46E-06	jaspar2016-GATA2-MA0036.1 /// jaspar2016-GATA3-MA0037.1 /// jaspar2016-YY1-MA0095.1 /// jaspar2016-ETS1-MA0098.1 /// jaspar2016-ZNF354C-MA0130.1
TNACTTGAACAGWK	4.51	6.46E-06	6.46E-06	jaspar2016-NKX2-8-MA0673.1 /// jolma2013-NKX2-8 /// jolma2013-NKX2-8-2

e4B. Motif enrichment for CpG in Model 2b (CpG sites associated with paternal BMI, within subset with maternal BMI >25.)				
**Motif**	**Score**	**P-value**	**FDR**	**Transcription factor binding sites**
YNCCYGGCACCGNN	6.83	8.24E-12	8.24E-11	
NCCYGCCCCCTCCBNN	6.06	1.34E-09	6.70E-09	jaspar2016-MZF1(var.2)-MA0057.1
CCCGGGCCCGGCBN	5.64	1.73E-08	5.78E-08	jaspar2016-TFAP2A-MA0003.1
NNCGCTGGGGGVNN	5.52	3.48E-08	8.70E-08	jaspar2016-MZF1-MA0056.1
HNCCTGTTAAAAYN	5.43	5.72E-08	1.14E-07	jaspar2016-MEIS1-MA0498.2 /// jaspar2016-MEIS2-MA0774.1 /// jaspar2016-MEIS3-MA0775.1 /// jolma2013-MEIS1 /// jolma2013-MEIS2-2 /// jolma2013-MEIS3
SNCAGGCGGAGGNN	5.33	9.64E-08	1.38E-07	jaspar2016-ETS1-MA0098.1
NCCCCGCCCCCANN	5.33	9.64E-08	1.38E-07	jaspar2016-MZF1-MA0056.1 /// jaspar2016-THAP1-MA0597.1 /// jaspar2016-ZNF740-MA0753.1 /// jolma2013-ZNF740 /// jolma2013-ZNF740-2
NNGGCCCCGCCCCN	4.93	8.21E-07	9.71E-07	jaspar2016-TFAP2A-MA0003.1 /// jaspar2016-SP1-MA0079.1 /// jaspar2016-KLF5-MA0599.1
NWAATGTCTTGARW	4.90	9.71E-07	9.71E-07	jaspar2016-MEIS1-MA0498.2 /// jolma2013-MEIS1
NRACGCCAGGCANR	4.90	9.71E-07	9.71E-07	jaspar2016-TFAP2A-MA0003.1 /// jaspar2016-NFIC-MA0161.1 /// jaspar2016-NFIX-MA0671.1 /// jolma2013-NFIX-2

© 2019 Noor N et al. *JAMA Network Open.*

eReferences

1. Cardenas A, Rifas-Shiman SL, Agha G, et al. Persistent DNA methylation changes associated with prenatal mercury exposure and cognitive performance during childhood. *Sci Rep.* 2017;7(1):288.
2. Agha G, Hajj H, Rifas-Shiman SL, et al. Birth weight-for-gestational age is associated with DNA methylation at birth and in childhood. *Clin Epigenetics.* 2016;8:118.
3. Tian FY, Rifas-Shiman SL, Cardenas A, et al. Maternal corticotropin-releasing hormone is associated with LEP DNA methylation at birth and in childhood: an epigenome-wide study in Project Viva. *Int J Obes (Lond).* 2019;43(6):1244-1255.
4. Heiss JA, Just AC. Identifying mislabeled and contaminated DNA methylation microarray data: an extended quality control toolset with examples from GEO. *Clin Epigenetics.* 2018;10:73.
5. Teschendorff AE, Marabita F, Lechner M, et al. A beta-mixture quantile normalization method for correcting probe design bias in Illumina Infinium 450 k DNA methylation data. *Bioinformatics.* 2013;29(2):189-196.
6. Triche TJ, Jr., Weisenberger DJ, Van Den Berg D, Laird PW, Siegmund KD. Low-level processing of Illumina Infinium DNA Methylation BeadArrays. *Nucleic Acids Res.* 2013;41(7):e90.
7. Johnson WE, Li C, Rabinovic A. Adjusting batch effects in microarray expression data using empirical Bayes methods. *Biostatistics.* 2007;8(1):118-127.
8. Du P, Zhang X, Huang CC, et al. Comparison of Beta-value and M-value methods for quantifying methylation levels by microarray analysis. *BMC Bioinformatics.* 2010;11:587.
9. Cardenas A, Allard C, Doyon M, et al. Validation of a DNA methylation reference panel for the estimation of nucleated cells types in cord blood. *Epigenetics.* 2016;11(11):773-779.
10. Bakulski KM, Feinberg JI, Andrews SV, et al. DNA methylation of cord blood cell types: Applications for mixed cell birth studies. *Epigenetics.* 2016;11(5):354-362.
11. Aryee MJ, Jaffe AE, Corrada-Bravo H, et al. Minfi: a flexible and comprehensive Bioconductor package for the analysis of Infinium DNA methylation microarrays. *Bioinformatics.* 2014;30(10):1363-1369.
12. Fagerberg L, Hallstrom BM, Oksvold P, et al. Analysis of the human tissue-specific expression by genome-wide integration of transcriptomics and antibody-based proteomics. *Mol Cell Proteomics.* 2014;13(2):397-406.
